# New Product Development in the Pharmaceutical Industry: Evidence from a generic market

**Published:** 2017

**Authors:** Nazila Yousefi, Gholamhossein Mehralian, Hamid Reza Rasekh, Mina Yousefi

**Affiliations:** a *Department of Pharmacoeconomics and Pharma Management, School of Pharmacy, Shahid Beheshti University of Medical Sciences, Tehran, Iran. *; b *Students’ research committee, school of pharmacy, Shahid Beheshti University of Medical Sciences, Tehran, Iran, *; c *Department of technology management, School of management and accounting, Azad University, Tehran, Iran.*

**Keywords:** New product development, Pharmaceutical industry, Analytical Hierarchy Process, Iran

## Abstract

In today’s competitive world, there are several strategies to deal with the fast changing environment, among which New product development (NPD) is a common strategy. However, almost half of the resources that companies devote to NPD are spent on products that may fail. This issue is particularly highlighted in the pharmaceutical industry mainly because of a long development-time, low success rate, high capital requirement, and market uncertainty. This study identifies critical success factors of NPD based on the relevant literatures and expert opinions in Iranian pharmaceutical industry, then prioritizes them using the methodology of multiple criteria decision making (MCDM) through analyzing 50 filled questionnaires structured based on the AHP (Analytical Hierarchy Process) approach. Although the NPD success factors seem the same in both generic and bio-generic pharmaceutical industries, the underlying factors and related sub-factors show the different importance in these two industries. However, this study reveal that, the company capabilities is the most important factor affecting new product development success in both pharmaceutical generic and bio-generic industry. The results of this study contribute to create baseline information for pharmaceutical industry especially Iranian pharmaceutical companies to be more effective in budget allocation on improving NPD success factors so that they can boost the success rate of NPD more effectively.

## Introduction

Such external pressures as globalized market in highly competitive environment(1), rapid technological changes, and short product lifecycles have made new product development important strategy for companies(2) in general and for dynamic industries such as pharmaceutical companies in particular. 

Historical data shows that the R&D expenditure in pharmaceutical firms, which is between 14% and 18% of their annual sales, is about five times more than average R&D expenditure in others industries (3). However, low R&D productivity, high R&D costs, tight regulation, low probabilities of technical success, unsure market, and limited qualified human resources(4) are driving the pharmaceutical industry to unprecedented challenges in new product development(5) while only 3 in 10 marketed drugs achieve revenues that match or exceed average research and development costs (6) and many pharmaceutical companiesʹ outputs have not been matched with their expenditures(7). This uncertainly in new product development(8) put pharmaceutical companies under pressure to produce successful products(9). Therefore, new product development success factors in pharmaceutical industry need more attention to reach acceptable level of financial return(10). Therefore, looking more closely to success factors of new products in this industry might help pharmaceutical industry achieve more successful new products.

Regardless of innovative level, which can be radical innovation by introducing new brand products or incremental by improving the existing products(11), NPD(New product development) is the most important determinant of sustained company performance(12). In this study NPD has been assigned to any changes in product portfolio(11) including macro level (new-to-market) or micro level (new-to-firm) new product(13).

As new product development is a high-risk and costly process(14) with significant failure rate(15), many researches focused on improving NPD by identifying several success factors(16). However, the success factors and their weights are varied in different industries(17). The NPD success factors depend on context specifications; in other words, resource allocation to the same success factors in different contexts may lead to different level of achievement. Hence, focusing on most relevant success factors can help companies to be more successful in new product development. Thus, evaluating the real affecting success factors in each context may bring great advantages for new product development (18).

Generic and bio-generic pharmaceutical companies have some key differences in NPD such as times and costs allocated to develop new products. Given longer clinical phases and longer regulatory approval periods in bio-generic companies, it takes a great deal of time if a biologic product could fulfill needed requirements to be launched to the market, while such requirements are not mandatory within generic companies. Moreover, both the cost of capital and costs related to product development are significantly higher in biopharmaceutical than traditional pharmaceutical firms(19). In addition, their market specifications are different; therefore, they would be considered two distinct contexts with different NPD success factors weights.

Introducing successful new products, which the growth and development of a firm depend on, requires technological knowledge and ability to transform it into valuable new products. In addition, complementary assets to facilitate the manufacturing, marketing, sales, and distribution of those products are required (20). This study aims to identify and prioritize the critical success factors of NPD in Iranian pharmaceutical industry --both generic and bio-generic pharmaceutical companies-- using AHP (Analytical Hierarchy Process) approach on 50 filled questionnaires. 

*Theoretical Framework*

Success factors of new product development are discussed in many studies(21). Senior manager commitment to new product development, qualified teams, proper internal and external relations and communications, innovative culture, and proper marketing support are some success factors which are expressed in previous studies(22). Based on Cooper et al. study in 1996, key success factors of new product development includes human capital, intellectual capital, organizational capital, relational capital, and organizational learning capability, where organizational capital includes their capabilities in launch, marketing, forecasting, and information gathering in a company(22). In another study, organizational capability of new product development categorizes into learning capability, R&D capability, manufacturing capability, marketing capability, strategic capability as well as resource allocation, and effective internal and external relations (networks) (23). 

In addition, Graner study in 2013, focused on improving NPD by using new structured methods and techniques (24), and Cooper and Edgett in 2008, classified new product development success factors into market environment, firm internal environment, organizational capability, NPD process, and level of new productʹs competitive advantage(25). The main categories of success factors in this study are retrieved from Brentani study in 2001 and cheng study in 2013, which are categorized to product-related, external context-related, and company-related factors(13, 26). Further discussion about each factor is elaborated upon hereunder. 

*Company-related factors*

Among company-related factors, managerial capabilities and management commitment to NPD projects are considered as two important factors in NPD success in literature(27). Top managersʹ supportive strategies toward innovation(28) as well as flexibility in different disciplines are also discussed as NPD success factors in literature(29). 

**Figure 1:**
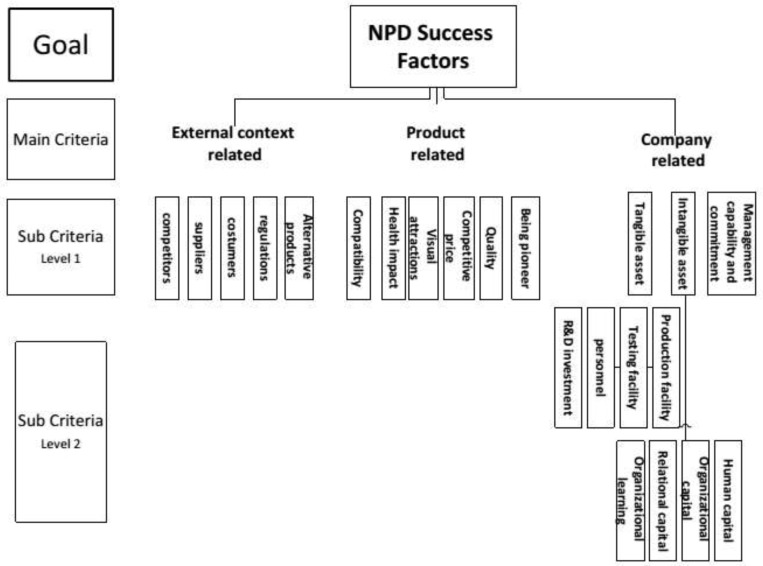
AHP hierarchy framework

**Table 1:** AHP nine-point scale

**Numerical Values**	**Definition**
1	Equally importance
3	Moderately more important
5	Strongly more important
7	Very strongly more important
9	Absolutely more important
2,4,6,8	Intermediate values

**Table 2:** Average Random Consistency Index (RCI).

**N**	1	2	3	4	5	6	7	8	9	10	11	12	13	14	15
**RCI**	0.00	0.00	0.58	0.90	1.12	1.24	1.32	1.41	1.45	1.49	1.51	1.48	1.56	1.57	1.59

**Table 3:** Related weight of main criteria

**Main criteria**	**Relative weight of Main criteria matrix (%)** **In ** **generic ** **pharmaceutical industry**	**CR**	**Relative weight of Main criteria matrix (%)** **In ** **bio-generic ** **pharmaceutical industry**	**CR**
Company-related	0.438	.0008	0.342	.0900
Product-related	0.395		0.318	
External context- related	0.167		0.341	

**Table 4:** Related weight of sub-criteria of company related factors

**Sub-criteria** **of** **company-related factors**	**Relative weight of Main criteria matrix (%)** **In** **generic** **pharmaceutical industry**	**CR**	**Relative weight of Main criteria matrix (%)** **In** **bio-generic** **pharmaceutical industry**	**CR**
Management capability and commitment	0.569	.0016	0.316	.0054
Intangible asset	0.219		0.338	
Tangible asset	0.213		0.346	

**Table 5:** Related weight of sub-criteria of product-related factors

**Sub-criteria** **of** **product-related factors**	**Relative weight of Main criteria matrix (%)** **In** **generic** **pharmaceutical industry**	**CR**	**Relative weight of Main criteria matrix (%)** **In** **bio-generic** **pharmaceutical industry**	**CR**
Being pioneer	0.180	.01	0.136	.01
Quality	0.328		0.247	
Competitive price	0.150		0.189	
Visual attraction	0.078		0.131	
Health impact	0.168		0.177	
Compatibility	0.095		0.120	

**Table 6:** Related weight of sub-criteria of external context-related factors

**Sub-criteria** **of** **External context-related factors**	**Relative weight of Main criteria matrix (%)** **In** **generic** **pharmaceutical industry**	**CR**	**Relative weight of Main criteria matrix (%)** **In** **bio-generic** **pharmaceutical industry**	**CR**
Alternative products	0.175	.0049	0.173	.0039
Regulations	0.211		0.179	
Customers	0.275		0.242	
Suppliers	0.113		0.203	
Competitors	0.226		0.204	

**Table 7:** Related weight of sub-criteria of tangible assets

**Sub-criteria** **of** **Tangible assets**	**Relative weight of Main criteria matrix (%)** **In** **generic** **pharmaceutical industry**	**CR**	**Relative weight of Main criteria matrix (%)** **In** **bio-generic** **pharmaceutical industry**	**CR**
Production facility	0.262	.030	0.240	.007
Testing facility	0.166		0.250	
personnel	0.139		0.202	
R&D investment	0.434		0.308	

**Table 8.** Related weight of sub-criteria of Intangible assets

**Sub-criteria** **of** **Intangible assets**	**Relative weight of Main criteria matrix (%)** **In ** **generic ** **pharmaceutical industry**	**CR**	**Relative weight of Main criteria matrix (%)** **In ** **bio-generic ** **pharmaceutical industry**	**CR**
**Human capital**	0.279	.0025	0.154	.0018
**Organizational capital**	0.258		0.323	
**Relational capital**	0.206		0.305	
**Organization learning **	0.258		0.219	

Tangible and intangible assets in companies are the next two company-related factors for NPD success. Currently more attention was paid to intangible assets as the important factor of innovation success(30). Intangible capital includes human capital, organizational capital, relational capital, and organizational learning(31). Personnel knowledge, expertise and behavior account for human capital and enable companies to develop new successful products(31). Organizational capital points to organization culture and its abilities for innovation and production. Itʹs formed by combination and coordination of different resources, lies in organizational routines, and generally consists of innovation capabilities, production capabilities, and marketing capabilities (32). In recent years, many organizations have been paying increasing attention to their social relationships with their various stakeholder groups(33). Relational capital or networking with universities, regulators, suppliers, and customers increases information capital in company and provides NPD resources for company more easily(34). Although organizational learning is a part of organizational capability, we consider it as a separate factor to show its importance in new product development. Organizational learning, which enables company by obtaining new knowledge from external and internal sources, makes a company more competitive in aspect of new product development advantages(35).

According to the resource-based view of firm, tangible assets are essential capability for product development. Accordingly, human resources, development of resources, testing resources, and the launch of resources significantly are committed to NPD projects and their financial success(36). Moreover, NPD is highly influenced by company investment capability in the development of projects (37). 

*Product-related factors*

Products are mostly developed to satisfy customersʹ needs (38); however, in the case of pharmaceuticals, not customersʹ needs but health system stakeholdersʹ interests should be considered. Therefore, the attribute of product health impact is taken in to account as a product-related factors in pharmaceuticals. Quality, in the same manner, which is essential feature for every new product(39), is strongly regulated in pharmaceutical industry.

Besides the quality of product, Khanna in 2012 mentioned that the development speed is the main success factor for pharmaceuticals (40). In the absence of other competitors, the first to market product can be efficiently marketed (41), and being pioneer is an extremely important competitive advantage for companies(7). However, even for the pioneers, the price proposed by companies for new medicine is one of the most important factors which determines the chance of new product for being welcomed by patients and health system(7). 

Finally, the new product compatibility with company knowledge and expertise can increase the new product success chance (42). In other word, company familiarity with developing and marketing similar medicines will increase its success rate in production and launch(43). 

*External context-related factors*

According to porterʹs theory in 1979, at least five external competition forces affect companiesʹ success. So, companies should go beyond rivaling current competitors by noting to customers, suppliers, potential entrants, and substitute products (44). More specifically, initially to increase success chance, to avoid entering high competition markets with high competitor entrance is recommended in literature (43). 

The next competition factor which should be considered is costumersʹ needs. Understanding costumer needs (14), translating it to value (45) and predicting consumer doubt toward new products (46, 47) are consider as crucial success factors for new product development in literature. Then, the supplier competition force is considered. As companies depend on a wide range of different supplier for new product development and production, suppliers can substantially affect the product success by charging higher prices, limiting quality, or services (48).

Finally, the threat of substitute products or alternatives is considered as an important competition force in new product success. Fast entering substitute products limits an industry’s potential profit from new products by placing a ceiling on prices and reducing market share (48) in general and in generic/bio-generic pharmaceutical industry in particular. 

In addition to effect of above mentioned competition forces(48, 49), success of new pharmaceutical products is highly affected by regulations (50). Regulatory bodies can affect the new product success through price setting, reimbursement or subsidizing policies,and licensing procedures(7). Furthermore, sufficient legal framework for patent right or market exclusivity plays a pivotal role in new product success through protecting new product value and motivating innovators to radical innovation (51).

*Iran pharmaceutical industry*

Pharmaceutical industry in Iran has a long history. For example, vaccines, as a modern pharmaceutical technology, was produced in Pasture institute in 1920 and Razi institute in 1925 (52). In 1979, Iran had approximately 40 pharmaceutical factories, most of which were the branches of international companies and were able to supply 30% of Iranian pharmaceutical market locally. As Iranian policy makers force international companies to leave the country after Islamic revolution in 1979, generic scheme as a new solution was introduced by Iranian experts. (53). Iran pharmaceutical industry, producing more than 95% of medicines consumed in Iran, has been very successful in improving accessibility and affordability of medicines (54); however, due to lack of proper investment in R&D activities and efficient investment in NPD , it is going to lose its competiveness in market. Generally, R&D investment in local industry and fundamental research is low so much so that (55) R&D activity in Iran pharmaceutical industry has been limited to new medicines formulation (56) during recent years. However, within the last decade, thanks to the presence of private sector, a great deal of fund has been invested in hi-tech biological for producing more innovative products (56). Due to different nature of generic and bio-generic industries, this study was designed to include both generic and bio-generic sectors. 

## Methods

The intensive literature review and intervening 11 experts having at least 5 years’ experience in pharmaceutical product development has led us to suggest a conceptual hierarchy chart for AHP (Analytical Hierarchy Process) consisting of new product success criteria and sub-criteria. Based on our comprehensive literature review, several success factors which are proposed by scholars for new product development are listed. Then we selected the fifty three most commonly cited critical success factors, and categorized them in three main domains; company-related, product-related, and external context-related. 

List of success criteria are screened during 11 interviews by experts who are highly involved in new product development during last 5 years to form final AHP hierarchy structure, including twenty eight success criteria in the main three domains. 

After that, we prioritizes criteria and sub-criteria using MCDM (multiple criteria decision making) methodology (57) through analyzing 50 filled questionnaires (23 in Bio-generic and 27 in generic companies) structured based on the AHP approach using pair-wise comparisons. AHP method was designed in early 1970 to show the way people actually think (58) it was widely applied in previous literature for multi-criteria decision making (59). AHP method, a multivariate analysis technique, helps decision makers reduce the entropy of subjective evaluations in complex decision making cases. By using AHP, decision problem is broken-down in to the multilevel decision attributes compared with each other and weights assigned to prioritize (60). AHP method not only assists decision-makers in achieving priorities and making optimal decisions, but gives them a clear rational validation of the choice as well. In addition, this method allows diverse or incomparable elements to be compared in a rational and consistent way (61). 

In this study, we used AHP method for prioritization of the success factors of new product development to assist decision makers to identify alternative course for action. Although a full AHP study is usually completed by alternative ranking, noting to study objectives, criteria ranking is the last step in this study. In this method, once the hierarchy structure is built, decision makers compared the various components placed in row to ones placed in column of matrices and vice versa to derive a numerical weight or priority of each element of the hierarchy (62). 

After pilot study, we found that our respondent were not convenient answering routine AHP tables, as a result of which the questionnaire was re-designed to visual scale comparison. Although tables with numerical scales are commonly used in the AHP for pair wise comparisons some new experiments confirm that the visual numerical scale suggested in 2006 by Zahir (63) can be more efficient and is preferable by respondents. 

At last, the judgments are analyzed using expert choice 11 software and analytical statistics including within and between the groups independence, deviation from consistency (CI: Consistency Index), and final weights (57) are considered in the acceptable level. 

*Hierarchy Structure*

AHP hierarchy including goal, criteria, and sub-criteria are structured in Figure 1. 

The goal of AHP hierarchy in this study is prioritizing the success factors of new product development in generic pharmaceutical industry. As it is shown in Figure 1, three main success criteria –company-related, product-related, and external context-related factors—are accounted for main goal in main level of hierarchy. In addition, there are 14 sub-criteria in the first level which totally achieve to 25 all levels. 

*Data collection and measurement*

First of all, main criteria and sub-criteria in the hierarchy are pair-wise compared by 23 experts in bio-generic companies and 27 experts in generic pharmaceutical companies using visual scaled AHP questionnaire. Experts have at least 5 years experience in new product development in pharmaceutical industry. In the next step, each pair of criteria was compared in nine-point Saaty scale (Table 1) to determine relative importance weights; So that, comparing objective i and objective j, give values 

aii = 1, aij = k , and aji = 1/k (62).

*Relative weights and consistency value analysis*

Calculation was made by expert choice 11 software to determine the relative weight of criteria and sub-criteria which is between 0 and 1. Then the ranking matrix of each level was calculated by pair wise comparisons and its consistency index (CI) and consistency ratio (CR) were measured to validate the results. As participants are often inconsistent in answering questions, consistency level of the estimated vector is measured in the following steps to determine reasonable consistency:

First for each matrix of order n, the relative weights and max are calculated. Then the consistency index for each matrix of order n is computed using the formulae CI = (max– n)/ (n –1). After that, the consistency ratio is calculated by the formulae CR = CI / RCI, where λ is the average consistency measure for all criteria, n is the number of criteria in each table, and RCI is a known as acceptable random consistency index for different matrix sizes which is shown in Table 2 (58).

Calculated CRs, which all are less than o.1, indicate that the pair-wise judgments are not just about random and are completely trustworthy.

## Results

As all the calculated CRs shown in Table 3-8 are less than 0.1, the pair comparisons made by the experts are assumed consistent and satisfactory for further analysis. Furthermore, table 3-8 shows the relative weights of the main criteria and their sub-criteria for new product success factors. Table 3 shows the most important criteria for both bio-generic and generic pharmaceutical companies that are company-related factors (0.342 and 0.438) followed by the external context (0.341), product-related factors (0.318) in Bio-generic, product-related characteristics (0.395), and external context-related factors (0.167) in generic industry (Table 3).

Among the sub-criteria of company-related factors which focus on company capabilities, tangible assets (0.346) is perceived to be the most important sub-criterion in bio-generic, followed by intangible assets (0.338), and management capability and commitment (0.316). Yet, in generic industry, management capability and commitment (0.569) shows the highest relative weight, followed by intangible assets (0.219), and tangible ones (0.213) (Table 4).

Among the sub-criteria of product-related factors which achieved the lowest importance weight for bio-generic, quality (0.247) is the most important success factor, followed by competitive price (0.189), productʹs positive impacts on health system (0.177), being pioneer in market (0.136), productʹs appearance and visual attractions (0.131), and compatibility with company experiences (0.120). Although in generic pharmaceutical industry the product quality similarly gains the first rank (0.328), the second ranking sub-criteria is time to market (0.180), followed by health impact (0.168), price (0.15), compatibility (0.095), and appearance (0.078) (Table 5).

Among the sub-criteria of external context-related factors or market-related factors by noting to Porter 5 competition forces, both bio-generic and generic industries, attention to customer needs is the first ranking external success factor (0.242 and 0.275) and competitors force is the second ranking one (0.204 and 0.226). These are followed by supplier sustainability (0.203), regulations (0.179), and entering new alternative products (0.173) in bio-generic companies and by regulations (0.211), entering new alternative products (0.175), and supplier sustainability (0.113) in generic ones (Table 6).

The Table 7 and 8 show the last level of AHP hierarchy, tangible and intangible sub-criteria. As shown in Table 7, company capability to invest on new product projects is the most important affecting factor in NPD success in both bio-generic (0.308) and generic industry (0.434). On the other hand, personnel shows the least importance in both industries (0.202 and 0.139). The second and third rankings assets in bio-generic industry are testing (0.25) and production facility (0.24); but in generic industry they still are production (0.262) and laboratory/testing facility (0.166) (Table 7).

Table 8 shows importance of intangible assets sub-criteria in bio-generic and generic companies. In bio-generic industry, organizational capital (0.323) including all the organization knowledge according to producing, testing, and marketing, obviously is the most critical factor to success followed by relational capital (0.305) including networking by universities, regulatory body, suppliers, and costumers. Organizational learning culture (0.219), which can promote new product development, achieved the next priority followed by human capital (0.154). In generic pharmaceutical industry the highest relative weight of intangible assets sub-criteria is dedicated to human capital (0.279), followed by organizational capital (0.258), organizational learning (0.258), and relational capital (0.206). 

## Discussion

New products can show R&D efforts in a company (64) and its ability to integrate internal and external competences to address rapidly changing environments (65). In the case of generic/bio-generic pharmaceutical producers, as the ‘R’ and ‘D’ activities have already been done by innovator companies, the development activities are just limited to replicate an accurate equivalent (66), thereby allocating less resources to R&D efforts and resulted in higher success rate. However, the failure risk in this industry is not negligible where about 27% of developed medicines by Iranian generic producers are never entered to the market (67). Therefore, this study aimed to increase new product success in Iranian bio-generic and generic pharmaceutical companies through identifying and prioritizing NPD success factors. 

Unlike generic medicines, in the case of bio-generic ones, the competition force is not high in the market and the bio-generics are still viewed as high technology products and the number of competitors is small in their market (40). This fact is parallel with this study result where the high value of biological products besides low competition intensity in their market place put the external context and product-related factors in the second and third priority. 

Generally speaking, regardless of product and context characteristics, we expect that the new bio-generic medicines have great chance to be successful in the market due to such companies act in a context in which a great deal of knowledge, capabilities, and complementary assets are imbedded (20). Despite the bio-generic context, the product-related factors is the second underlying factor in NPD success in generic companies. 

According to literature, it should be taken into the account that different company assets have different value for new product development in different industries, as a result of which, some resources should be considered more critical than others (32) in each industry. As bio-generic companies are knowledge-based companies which mainly focus on R&D efforts, intellectual capital should play a critical role in such companies. However, tangible assets achieve the highest priority for new product development in Iranian bio-generic industry. According to Dadfar study in Iranian pharmaceutical industry, the common source of new technologies in Iranian company is mostly transferred from external sources (68). With this in mind, tangible assets achieve higher weight than intangible assets and management capabilities. As generic companies already have proper technological platform, managerial and intangible asset show higher importance for managing current technology for new product development. This is fairly consistent with literature emphasizing the effect of management capability and commitment on NPD success 

(27). As the main goal of generic pharmaceutical companies is to produce products with low price in short development-time, they mainly focus on production and sales. Hence, management capability has gained the highest rank in these companies. 

Among sub-criteria of tangible assets, the higher priority of production facility in generic industry interestingly changed to laboratory/testing facility in bio-generic industry. This can be justified by complexity of testing procedures in bio-generic which cannot be easily outsourced to external laboratories in Iran. Alongside difficult sample transferring, overseas contracts for laboratory testing are very time-consuming and costly for these companies. Considering intangible assets sub-criteria, organizational capital and relational capital show higher priorities in bio-generic companies. However, the two first priorities in generic companies were human capital and organizational capital. 

Organizational capital which in both generic and bio-generic companies grant high priority is also remarked in literature as a main success factor in NPD (69, 70), and has been defined as the knowledge, human skills, and structural capital, which are combined into a system for producing satisfying products. Relational capital not only can improve product success through networking but also can affect it through establishing a business and political tie by stakeholders. 

Following costumer and competitor which are the first two important factors among external context-related factors, regulatory issues show high importance in generic industry; however, it does not show the same importance in bio-generic industry where is placed at the fourth rank among external forces. It can be justified by the supportive approach of government to bio-industry which is reflected in related policies and regulations. In addition, as the development of alternative bio- medicines by competitors takes a lot of time and cost, concerning about coming alternative products (14) in this context is the last important factor. However, the last issue among external forces is suppliers force in generic pharmaceutical industry in that a variety of suppliers are available for such companies all over the world. 

Similar to cross national study which expresses the quality as the main affecting factor in new product success (71), this study specifies quality as the most important sub-criteria of product-related factors, by both bio-generic and generic pharmaceutical experts. Conversely, competitive price shows the low importance in both bio-generic and generic industry. As the bio-generic are significantly cheaper than their original competitor, they are almost reimbursed and subsidized by health system in Iran. The price of these products in comparison to their competitors is sufficiently low to attract attention of both consumer and health system(43). Moreover, in the case of generic medicines, the fixed minimum reimbursed price accepted by payers in Iran resulted in an atmosphere in the market in which pharmaceutical pricing cannot play a pivotal role in competitiveness. In that, the policy issued by payers imposes pharmaceutical companies to set their products prices in a same fashion.

Finally, time-to-market shows higher priority in generic industry rather than biopharmaceutical industry. Being pioneer is important for new products succession generally and for new products in a market with low legal protection particularly. Legal inadequacy in protecting new products right either patent or market exclusivity and dysfunctional competition in an intensive market remarkably enhance the importance of NPD speed (51) in Iranian generic market. However, it is also important for bio-generic companies to release their products to the markets much faster than their rivals. 

In conclusion, this study recommends that generic pharmaceutical companies should pay higher attention to their new product characteristics especially quality, price, and time to market rather than external context characteristics such as suppliers and alternatives products. Conversely, the external context characteristics especially customers, suppliers, and competitorsʹ specifications need higher attention in bio-generic pharmaceutical companies.

To improve company capabilities for developing new product, the results of this study strongly suggest that it would be more beneficial for generic companies to pay their highest attention to management capability. 

*Implication*

Although the NPD success factors are similar in different industries, they are diversely weighted in different contexts. More specifically, diversity of technologies, different level of competition, and capabilities of industries can indeed change the priority of success factors. The mean investment on R&D which is about 7% of their sales in generics medicine industry (72), is around 16% in innovator companies (73), while the average of R&D cost is about 1% in Iranian pharmaceutical industry (74). Therefore, the efficient allocation of funds on NPD success factors would highly recommend in developing courtiers such as Iran with the limited R&D budget. 

Hence, each industry by knowing its own success factors and their related weights can allocate their budget in an efficient manner in order to boost significantly the success rate of new products.

*Limitations*

Different research contexts could provide additional useful information to both critical success factors and their ranking. In addition, different research methodology may show different weight of success factor. The main research opportunity exists in testing the results by different research methodology such as ANP (The analytic network process).
